# Task-Specific Effects of tDCS-Induced Cortical Excitability Changes on Cognitive and Motor Sequence Set Shifting Performance

**DOI:** 10.1371/journal.pone.0024140

**Published:** 2011-09-01

**Authors:** Jorge Leite, Sandra Carvalho, Felipe Fregni, Óscar F. Gonçalves

**Affiliations:** 1 Neuropsychophysiology Laboratory, CIPsi, School of Psychology (EPsi), University of Minho, Braga, Portugal; 2 Neuromodulation Laboratory, Spaulding Rehabilitation Hospital, Harvard Medical School, Boston, Massachusetts, United States of America; Katholieke Universiteit Leuven, Belgium

## Abstract

In this study, we tested the effects of transcranial Direct Current Stimulation (tDCS) on two set shifting tasks. Set shifting ability is defined as the capacity to switch between mental sets or actions and requires the activation of a distributed neural network. Thirty healthy subjects (fifteen per site) received anodal, cathodal and sham stimulation of the dorsolateral prefrontal cortex (DLPFC) or the primary motor cortex (M1). We measured set shifting in both cognitive and motor tasks. The results show that both anodal and cathodal single session tDCS can modulate cognitive and motor tasks. However, an interaction was found between task and type of stimulation as anodal tDCS of DLPFC and M1 was found to increase performance in the cognitive task, while cathodal tDCS of DLPFC and M1 had the opposite effect on the motor task. Additionally, tDCS effects seem to be most evident on the speed of changing sets, rather than on reducing the number of errors or increasing the efficacy of irrelevant set filtering.

## Introduction

The capacity for shifting cognitive processes, such as shifting attention, learning or simply adapting to new environmental changes, is one of the most distinctive human abilities. In this study, set is defined as the property of the stimulus that is relevant for the task [1], namely color, shape or the specific motor sequence that the participant has to sequentially reproduce (A or B). Set shifting ability may be defined as the capacity to switch between sets (e.g. from color to shape in two consecutive trials in the cognitive task, or from A to B in the motor sequence task) while the goal is maintained [2], or the capacity to move back and forth between mental sets or tasks [3]. Set shifting has been associated with executive control [4], involving processes such as planning, goal-directed behavior, and cognitive flexibility [5].

Set shifting ability is thought to involve a highly engaged network within the brain that consists of several cortical and subcortical structures. Neuroimaging studies have reported evidence of increased activation of the pre-frontal cortex (PFC) in set shifting tasks [6], [7]. Impairments in set shifting have been shown in patients with damage to the prefrontal cortex [4], [8], [9], particularly in the left hemisphere [10].

Although higher hierarchical cognitive functions, such as set shifting, are associated with neocortical areas, there is increasing evidence that subcortical structures, such as the basal ganglia, are also involved and operate, particularly, as gating mechanisms [11], [12], [13]. In fact, there are an impressive number of inputs from sensory, premotor and motor areas, as well as from association areas in the frontal, parietal, medial, and temporal cortices to the basal ganglia [14], [15], [16], [17], [18]. And there is clear evidence of set-shifting deficits in clinical disorders associated with basal ganglia dysfunction, such as Parkinson's [8], Huntington's [19], eating disorders [20], and Obsessive-compulsive disorder [21], [22]. Taken together, the data from the literature suggest that a distributed, but highly engaged neural network is involved in set shifting.

As previous research has shown, modification of the excitability of the left dorsolateral prefrontal cortex (DLPFC) and the primary motor cortex (M1) can significantly change behavior associated with these areas [23], [24], [25]. The aim of this study is to test if anodal, cathodal and sham transcranial direct current stimulation (tDCS) in DLPFC and M1 can modulate set shifting tasks. Objectively, we aim to test if the effects of tDCS on performance are due to changes in the speed of processing, shift costs, alterations in irrelevant set filtering or in the number of errors.

## Results

None of the participants in this study reported mood alterations due to stimulation or have experienced any adverse effects. In the sham condition, participants reported a tingling sensation, as in the active tDCS conditions. The present section discusses each task independently, analyzing them with regard to the following: (i) the Reaction Time (RT) required to perform the task; (ii) the number of errors in task performance; and (iii) the time difference between shift and no shift trials (i.e., shift cost). Additionally, task characteristics were analyzed to test if the task revealed shifting and filtering differences. Because of the limited power of this study to conduct a model including all the factors, we show an additional exploratory analysis at the end of this section testing the interaction between task and the polarity of tDCS.

### Cognitive Task: (i) Reaction Time

#### a. Analysis of the task

**Figure 1:**
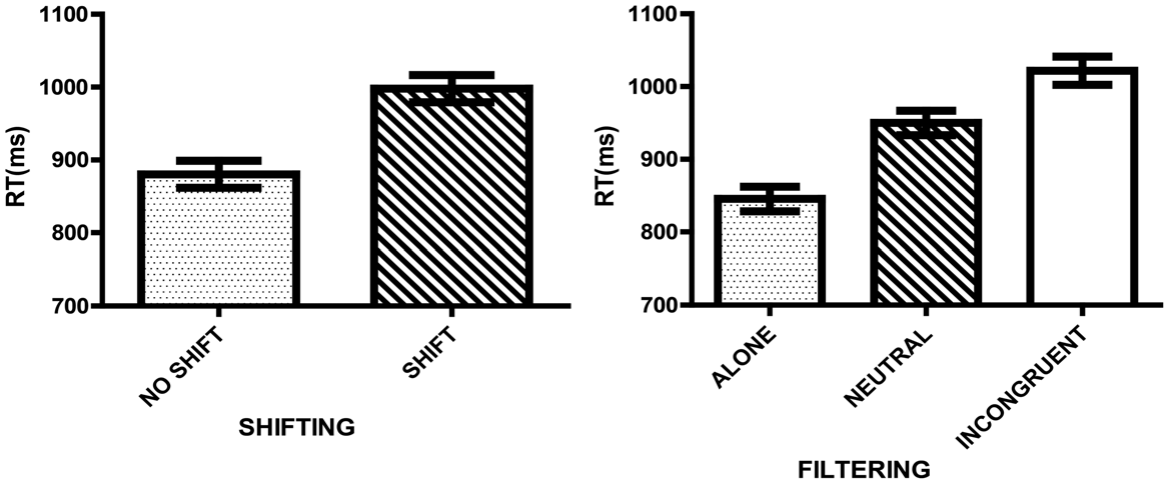
Performance as indexed by RT in the Cognitive Task. Columns represent the MEAN and the bars the SEM for the RT (95% CI) in the Cognitive Task. Shifting: No shift trials represent the RT when the set remained the same (e.g. color – color) while Shift represents the RT when there were changes in the set (e.g. shape – color). Filtering: For Alone there was no competing set; for Neutral there was an irrelevant competing set, and for Incongruent there was a relevant competing set.

There was a shifting effect (F (1,28) = 69.174, p<.001, η_p_^2^ = .712). As expected in the No Shift condition (M = 880.208, SE = 18.518), participants showed significantly smaller RTs than in the Shift condition (M = 997.874, SE = 18.697) (p<.001). In terms of filtering competing sets, RTs were significantly different across all conditions (F(2,56) = 336,260, p<.001, η_p_^2^ = .923) showing a gradation effect: Alone (M = 845.368, SE = 16.712)<Neutral (M = 950.019, SE = 16.871)<Incongruent (M = 1021.736, SE = 19.274) (p<.001) (see Fig. 1).

#### b. Effects of polarity

**Figure 2:**
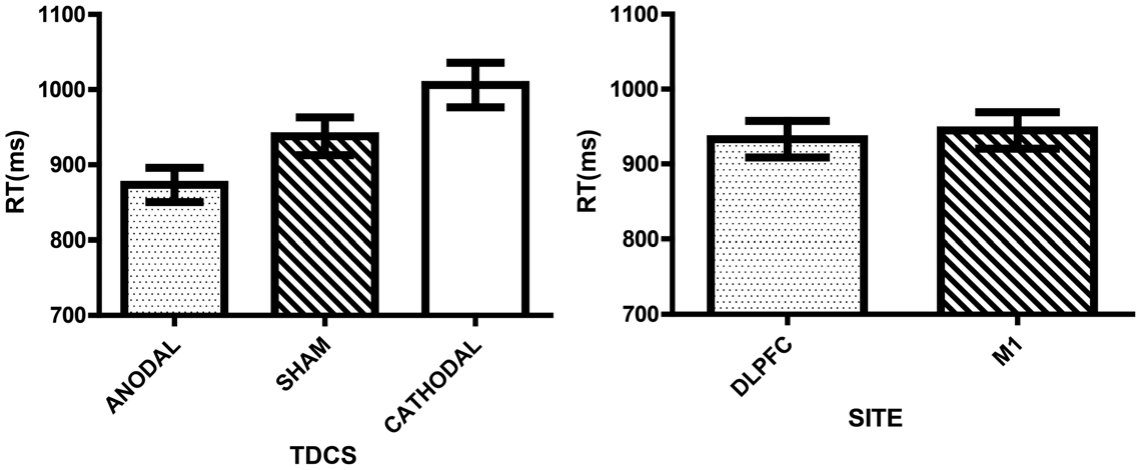
Performance as indexed by RT: Polarity and Site effects. Columns represent the MEAN and the bars the SEM for the RT (95% CI) in the Cognitive Task. tDCS: Represents the polarity effects in RT. Site: Represents the estimates of the RT per site of stimulation.

There was a significant effect of tDCS on task performance (F(2,56) = 7.763, ε = .834, p = .002, η_p_^2^ =  .217). Anodal stimulation (M = 873.040, SE = 22.835) decreased RTs significantly when compared to either sham (M = 937.937, SE = 25.172) (p = .046) or cathodal stimulation (M = 1006.145, SE = 29.578) (p = .004). Cathodal stimulation was not significantly different from sham (p = .226) (see Fig. 2).

There was no significant interaction between polarity and the filtering of the competing set(F(4,112) = 1.850, p = .124, η_p_^2^ = .062).

#### c. Site effects

There were no significant effects associated with the site of stimulation (F(1,28) = .115, p = .737, η_p_^2^ = .004).

### Cognitive Task: (ii) Number of errors

#### a. Analysis of the task

There were no significant differences in the number of errors in terms of shifting (F(1,28) = 1.334, p = .258, η_p_^2^ = .045).

 There were differences in terms of the number of errors due to set filtering (F(2,56) = 11.912, p<.001, η_p_^2^ = .298). Incongruent filtering (M = 1.939, SE = .262) significantly increased the number of errors compared to both Neutral (M = 1.161, SE = .188) (p = .002) and Alone (M = 1.194, SE = .191) (p = .002). There were no differences between Alone and Neutral filtering (p = 1,000) (see table 1).

**Table 1:** Mean Number of errors and percentage of correct responses for the cognitive task.

	COGNITIVE (Error)						COGNITIVE (Correct Responses %)					
	ANODAL		SHAM		CATODAL		ANODAL		SHAM		CATODAL	
	NS	S	NS	S	NS	S	NS	S	NS	S	NS	S
**DLPFC**	4,53 (5,25)	5,47 (4,70)	3,93 (3,22)	4,60 (3,76)	4,60 (5,26)	4,47 (5,33)	93,70 (7,29)	92,41 (6,53)	94,54 (4,47)	93,61 (5,22)	93,61 (7,31)	93,80 (7,40)
**M1**	4,13 (3,07)	6,20 (5,10)	4,73 (5,24)	7,20 (7,59)	4,67 (3,94)	6,47 (6,14)	94,26 (4,26)	91,39 (7,09)	93,43 (7,28)	90,00 (10,54)	93,52 (5,47)	91,02 (8,53)

NS- No Shift Trial; S – Shift Trial. The values are means and the standard deviation is showed in brackets. On the left, table 1 represents the total number of errors for the cognitive task. On the right, the percentage of correct responses to the same task is showed. Percentage of correct responses is included in order to provide a comparison between tasks.

#### b. Effects of polarity

There were no differences in terms of the number of errors due to polarity (F(2,56) = .274, p = .762, η_p_^2^ = .010). There was no significant interaction on the number of errors between polarity and the filtering of the competing set (F(4,112) = 1.928, p = .111, η_p_^2^ = .064).

#### c. Site effects

 There were no significant differences in the number of errors associated with the site of stimulation (F(1,28) = .028, p = .869, η_p_^2^ = .001).

### Cognitive Task: (iii) Shift costs

#### a. Analysis of the task

 There were differences in terms of Shift costs due to set filtering (F(2,56) = 34.979, p<.001, η_p_^2^ = .555). Incongruent filtering (M = 58.885, SE = 18.965) decreased the Shift costs significantly compared to both Neutral (M = 162.310, SE = 13.005) (p<.001) and Alone (M = 131.802, SE = 15.269) (p = .001). In addition, Alone filtering showed significantly smaller Shift costs than Neutral (p = .031).

#### b. Effects of polarity

 There were no significant differences in terms of Shift costs due to polarity (F(2,56) = 1.407, p = .253, η_p_^2^ = .048).

There was no significant interaction in terms of Shift costs between polarity and the filtering of the competing set (F(4,112) = .373, p = .827, η_p_^2^ = .013).

#### c. Site effects

There were no significant effects associated with the site of stimulation (F(1,28) = 1.249, p = .273, η_p_^2^ = .043).

### Motor Task: (i) Reaction Time (RT)

#### a. Analysis of the task

**Figure 3:**
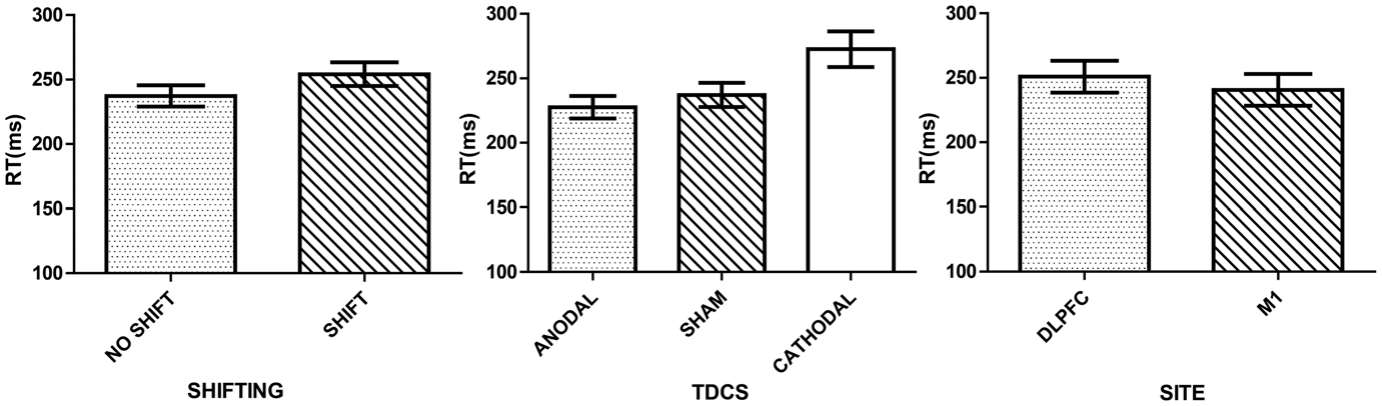
Performance as indexed by RT in the Motor Task. Columns represent the MEAN and the bars the SEM (95% CI) for the RT in the Motor Task. Shifting: No shift represents the RT when the motor sequence remained the same (e.g., AA or BB), while Shift represents the RT when there were changes in the motor sequence set (e.g., AB or BA). tDCS: Represents the effects of polarity on performance. Site: Represents the estimates of the RT per site.

There was a shifting effect (F(1,28) = 49.043, p<.001, η_p_^2^ = .637). As expected in the No Shift condition (M = 237.297, SE = 8.256), participants showed significantly smaller RTs than in the Shift condition (M = 254.160, SE = 9.300) (p<.001) (see Fig. 3).

#### b. Effects of polarity

There was a significant effect of tDCS on task performance (F(2,56) = 8.945, ε = .740, p = .002, η_p_^2^ = .242). Cathodal stimulation (M = 272.604, SE = 13.793) increased RTs significantly when compared to both sham (M = 237.049, SE = 9.370) (p = .012) and anodal stimulation (M = 227.531, SE = 8.705) (p = .009). Anodal stimulation was not significantly different from sham (p = .697).

#### c. Site effects

There were no significant effects associated with the site (F(1,28) = .357, p = .555, η_p_^2^ = .013).

### Motor Task: (ii) Number of errors

#### a. Analysis of the task

There were significant differences in the number of errors in terms of shifting (F(1,28) = 9.833, p = .004, η_p_^2^ = .260). The No Shift condition (M = .278, SE = .084) showed significantly fewer errors than the Shift condition (M = .722, SE = .188) (see table 2).

**Table 2:** Mean Number of errors and percentage of correct responses for the motor task.

	Motor (Error)						Motor (Correct Responses%)					
	ANODAL	SHAM		CATODAL		ANODAL		SHAM		CATODAL	
	NS	S	NS	S	NS	NS	NS	S	NS	S	NS	S
**DLPFC**	0,07 (0,26)	0,73 (0,96)	0,27 (0,59)	1,27 (2,37)	0,33 (0,62)	0,40 (0,91)	99,83 (0,65)	98,17 (2,40)	99,33 (1,48)	96,83 (5,94)	99,17 (1,54)	99,00 (2,28)
**M1**	0,27 (0,59)	0,53 (0,83)	0,27 (0,59)	0,27 (0,59)	0,47 (1,36)	1,13 (2,88)	99,33 (1,48)	98,67 (2,08)	99,33 (1,48)	99,33 (1,48)	98,83 (3,39)	97,17 (7,19)

NS- No Shift Trial; S – Shift Trial. The values are means and the standard deviation is showed in brackets. On the left, table 2 represents the total number of errors for the motor task. On the right, the percentage of correct responses to the same task is showed. Percentage of correct responses is included in order to provide a comparison between tasks.

#### b. Effects of polarity

There were no differences in terms of the number of errors due to polarity (F(2,56) = .224, ε = .770, p = .742, η_p_^2^ = .008).

#### c. Site effects

There were no significant differences in the number of errors associated with the site (F(1,28) = .008, p = .931, η_p_^2^ = .000).

### Motor Task: (iii) Shift costs

#### a. Effects of polarity

There were no differences in terms of Shift costs due to polarity (F(2,56) = 1.491, p = .234, η_p_^2^ = .051).

#### b. Site effects

There were no significant effects associated with the site of stimulation (F(1,28) = 1.205, p = .282, η_p_^2^ = .041).

### Interaction testing between task and tDCS polarity

 There was a significant effect of the polarity interacting with the task performance in terms of RTs (F(2,56) = 5.015,p = .010, η_p_^2^ = .152). No statistically significant interactions between tasks and tDCS polarity were found in terms of shift costs (F(2,56) = .815, p = .448, η_p_^2^ = .028) and the percentage of correct responses (F (2,56) = .100, p = .905, η_p_^2^ = .004).

## Discussion

The present study tested the effects of tDCS-induced cortical excitability changes (anodal, cathodal and sham tDCS) in DLPFC and M1 on two different set shifting tasks (cognitive and motor). For the cognitive task, anodal stimulation was found to increase performance as indicated by an RT decrease compared to both sham and cathodal conditions. Although cathodal tDCS decreased overall performance, there was no statistically significant difference when compared to sham tDCS. However, in the motor task, cathodal stimulation significantly decreased performance when compared to sham and anodal stimulation.

One important finding is that these results are independent of the stimulation site, suggesting a non-specific site effect probably due to interactions among the several neural networks that have been shown to be activated when performing set shifting/task switching tasks [26], [27], [28], [29], [30], [31]. Task switching research has demonstrated that performing one task and then another could activate a common frontal parietal network [32]. Moreover, both motor and executive functioning areas could be responsible for distinct cognitive processes involved in a broader cognitive control process [33]. However, that does not entirely explain the tDCS effects found in this study, especially the task polarity interaction.

One hypothesis is that the effects are dependent on the level of activation of this network. In other words, for the cognitive task, in which the demand on motor systems is less intense, anodal tDCS was able to enhance performance as the system was likely engaging a more reduced neural network as compared to that engaged by the motor task. In fact, for the motor task, because the co-activation of motor and executive areas was likely more intense, an increase in activity induced in only one area was not sufficient to enhance performance. On the other hand, the cathodal-induced excitability decrease in motor or prefrontal areas was associated with a performance reduction in the motor task due to activity reduction in one region of this highly engaged network required for performance of both tasks.

There are also alternative hypotheses to explain our results. For instance, the lack of specific effects might be explained by the lack of focality of the tDCS. In this scenario, DLPFC tDCS induced similar effects as M1 tDCS due to the lack of focality. However, modeling and behavioral studies tend not to support this alternative explanation, as they show that the peak of the current is induced under the electrode [34], [35] and also that DLPFC and M1 tDCS induce different behavioral effects [24], [36], [37]. Alternatively, as the “reference” electrode was positioned over the contralateral supraorbital area, it is also possible that this electrode exerted an effect on our results. This hypothesis arises because Brodmann Area (BA) 10 has been associated with these particular types of tasks [38], [39], [40] and because tDCS studies have shown effects on cognitive processing induced by that particular site [41]. Future studies need to assess other electrode montages to rule out this effect, namely, by using extra-cephalic reference electrodes. Using smaller electrodes will also be a future option for testing non-specific results.

 In terms of the filtering of irrelevant information, the pattern found in this study was Alone<Neutral<Incongruent, which is consistent with previous studies [27]. The explanation that has been provided for this is that as the distracting set gets more challenging, there is an increased demand on filtering [27]. This study shows that anodal and cathodal tDCS both modulate cognitive and motor tasks. They had a consistent effect on results independent of the site of stimulation (anodal improved and cathodal decreased task performance), suggesting that the cortical stimulation is modulating this highly engaged network involved in set shifting.

There were no specific effects of tDCS on the filtering of irrelevant information or on Shift costs. There were also no errors related to tDCS. The error effects found were related to shift or filtering conditions, and are being interpreted as more demand on resources due to normal task performance.

Future research using an fMRI paradigm should explore the assumption that cortical tDCS could interfere with shift ability by affecting this highly engaged network (with cortical and eventually subcortical processing) to establish the cortical tDCS effects and possible cortical-striatum interactions. In addition, future studies should also explore neuromodulation of cortical-subcortical activity in different pathologies with set shifting impairments, namely Obsessive-compulsive disorder, eating disorders, Parkinson's and Huntington's disease, as well as in aging.

 Future research should also focus on the effects of tDCS on dopamine receptors using these set shifting tasks, as the administration of D2 antagonists in healthy subjects [42] showed an effect on set shifting similar to the one found in this study with cathodal stimulation.

One of the limitations of this study was the lack of statistical power to include all the factors in a full multifactorial analysis. Thus some of the results need to be seen as exploratory and need to be confirmed with larger sample sizes. Also future research should apply tDCS during the actual task, in order to compare the results from learning phase to actual performance, as there could be specific learning phase effects [43].Also, the cognitive task took longer to perform than the motor one. This time difference found in performance between tasks may be a limitation of the present study. Future studies should match the duration of the cognitive and the motor task (possibly by establishing a time limit rather than number of trials). In conclusion, the present study found that both anodal and cathodal tDCS can modulate a cognitive–motor task. The non-specific site effects could be related to an interaction within this neural network, to the network demand involved in these two tasks, or to the enrollment of the right supraorbital in this highly engaged network. Finally, a single session of tDCS to the left DLPFC or to M1 (or the right supraorbital) seemed to have a greater result on the speed of changing sets than on Shift costs, either by reducing the number of errors or by increasing the efficacy of irrelevant set filtering.

## Methods

### Study overview

In the present study, we tested the effects of anodal, cathodal and sham stimulation of DLPFC (F3 electrode site) and M1 (C3 electrode site) in separate experiments on two different set shifting tasks, one motor and one cognitive [27]. Participants were divided into two groups of fifteen, namely, to receive tDCS on DLPFC or M1. They performed both tasks in three distinct sessions (one per polarity of tDCS).

**Figure 4:**
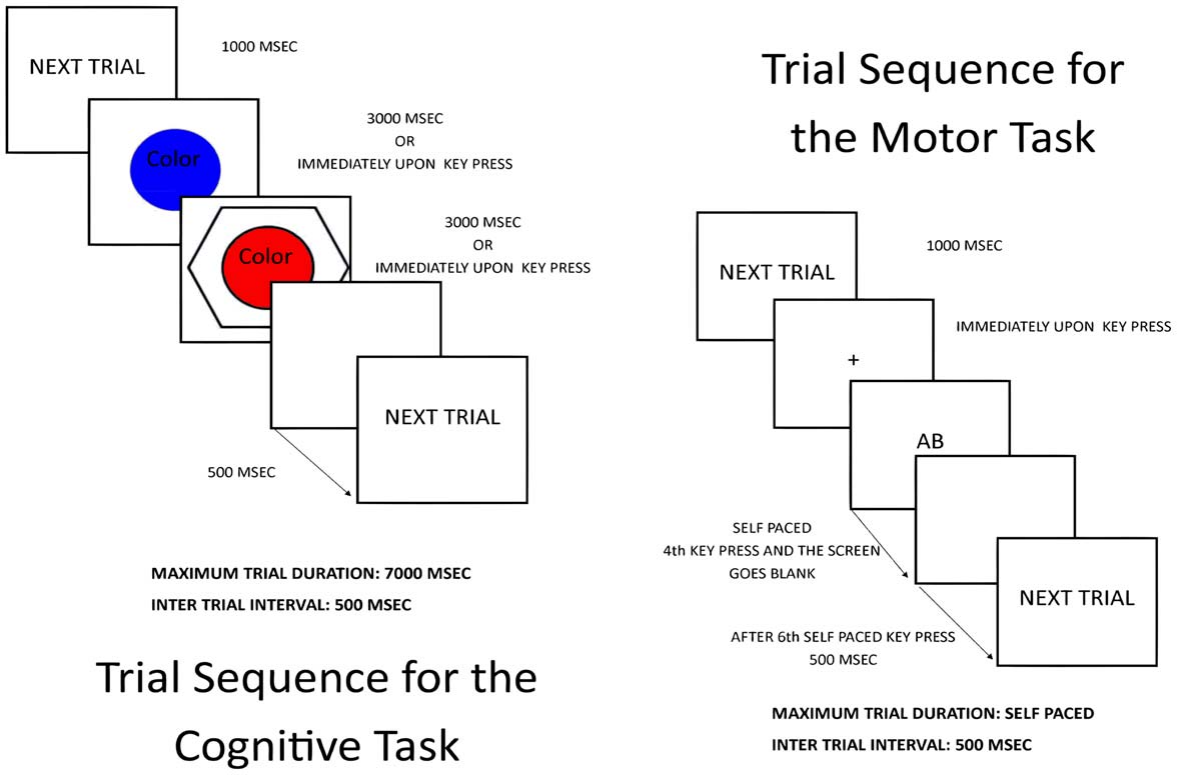
Schematic Representation of the tasks used in the experiment. Each trial started with a next trial message on the center of the screen. Cognitive task: In this task, each trial consisted of a pair of stimulus. The set that the participant was required to respond could remain the same (e.g. Color S1 and Color S2) or could be different (e.g. Shape S1 and Color S2). Motor Task: In each trial a pair of letters appeared on screen (each letter represents a motor sequence of three key presses previous learned).

In the cognitive set-shifting task, the participants were instructed to respond either to color or shape having previously associated two colors and two shapes to the same response buttons: 1 and 2. In the motor set-shifting task, there were two sequences of three keystrokes that they should perform using only the index finger of the right hand. These two sequences were performed in response to the stimulus that appeared randomly on the screen. The stimulus consisted of a pair of letters, each one associated with a three-keystroke sequence learned previously. In both tasks, using two consecutive stimuli, the set either remained the same (e.g. color-color or same letter) or was different (e.g. color-shape or different letter) (as depicted in Fig. 4).

### Participants

Thirty university student volunteers participated in the study. All of the participants were healthy, with normal or corrected-to-normal visual acuity, with a score on the Edinburgh Handedness Inventory (EHI) [44] of ≥80 (right handed) and without present or past history of neurological or psychiatric disorder. Participants were excluded if any medication or psychotropic drugs had been used during the 4 weeks prior to the study. Participants were advised to avoid alcohol, cigarettes and caffeinated drinks on the day of the experiment, and none reported fatigue due to insufficient sleep.

### Ethics Statement

All of the participants gave their written informed consent prior to their inclusion in the study. The study was approved by the local ethics committee Centro de Investigação em Psicologia (CIPsi) and was in accordance with the Declaration of Helsinki.

### Procedure

#### a. Cognitive Task

Two colors, red and blue, were assigned, respectively, to response keys 1 and 2. A third color, green, was not assigned to any key and acted as a neutral distractor. The colors appeared as a circle with a diameter of approximately 2.0° of visual angle (viewing distance was approximately 1 meter). Two shapes, triangle and square, were assigned to the same two response keys, 1 and 2, respectively. A third shape, hexagon, was not assigned to any key and functioned as a neutral distractor. The shapes appeared as non-filled figures outlined with a line weight of three pixels. In height and width, each shape subtended a visual angle of approximately 3.8° in width and 3.2° in height at 1.5 meters of viewing distance.

Each stimulus figure was accompanied by a set instruction (the word *Color* or *Shape*) informing participants in which set they were required to respond. The word appeared on the fixation point at the center of the screen. The word was approximately 0.6° of visual angle in height and 1.5° in width.

Participants were instructed that they should associate the two colors and the two shapes with the same response buttons (1: red or triangle; 2: blue or square). They were also instructed that a word *Color* or *Shape* would appear on the center of the screen that would identify the set in which they were required to respond.

In all sessions, the participants performed the training phase that lasted 5 min. During this phase, after the participant responded there was a 500 msec interval before the next trial began with the “Next trial” message. If, for either stimulus, the subject pressed any key other than the correct response, the incorrect response was immediately followed by a 1 sec error message before the program continued (during the task phase there was no error message). There were a total of 30 trials that the participant should correctly answer in order to finish the training phase. The task consisted of two paired stimuli, S1 and S2, about which the participant was asked to make the same judgment. Immediately after responding to S1 (or automatically after 3000 msec if no key was pressed), S2 appeared on screen. If S1 and S2 had the same set (color or shape), it was considered a no shift trial. Instead, if S1 and S2 differed in set (e.g. S1 color and S2 shape), it was considered a shift trial.

There were also three types of filtering conditions, namely: Alone, Neutral, and Incongruent. For the Alone filtering condition, a color or shape appeared without any distractor from the other set. For the Neutral filtering condition, a color or shape appeared with the neutral distractor (green or hexagon) from the other set. For the Incongruent filtering condition, a color or shape appeared with the distractor that had the alternative response key associated with it. Alone and Neutral consisted of four possibilities: Alone (red, blue, triangle, square); Neutral (red and hexagon, blue and hexagon, triangle and green, square and green). The Incongruent condition only consisted of two possibilities (red and square, blue and triangle). There was no congruent condition (e.g. red and triangle, blue and square). For the Neutral and Incongruent figures, the circular color patch appeared centered within the outlined shape.

These conditions were designed to test filtering effects on response selection, i.e., the competing set that should be irrelevant to task performance. The Alone condition had no competing set, the Neutral condition had a neutral distractor competing set, and the Incongruent condition had an incongruent response competing set.

Stimuli were presented on a white background on a total of 144 trials, and each experimental condition was fully randomized and had the same probability.

#### b. Motor Task

In this task, the central fixation point presented at the beginning was replaced by a pair of letters (AA, BB, AB, BA). Both the fixator and the pair of letters had the height and width of approximately 0.6° of visual angle. The letters and the fixation cross were black on a white background.

A keypad with three response keys was placed in front of the participant, allowing access with the dominant hand. The three keys on the pad were labeled 1, 2, and 3 and were arranged in an equilateral triangle approximately 1.5 cm apart from one another. The letters A and B were associated with the sequences 1–2–3 and 1–3–2, respectively, in the training phase, that consisted of twenty four correct self-paced trials. In the training phase, the order in which the participant should perform the cognitive and motor tasks was randomized and counterbalanced across participants and sessions, and both of them were performed during the last five minutes of active stimulation. The task consisted of two different, previously learned sequences: A (123) and B (132), and then reproducing them in pairs, using only the index finger, following the instruction on the screen. Each trial consisted of a total of six self-paced key presses.

Each trial had the following sequence: a fixating cross in the middle of the screen that should elicit a response with a key marked with a cross. This key was at the center of the equilateral triangle formed by the other response keys (1, 2 and 3). This was chosen because it allowed for experimental control of the starting point of each experiment. 1000 msec after pressing the key with the cross, the two letters appeared on the screen. Those two letters represented the two sequences that would be required for the participant (e.g., BA) and remained on the screen until the 4th key of the sequence (first of the second sequence) was pushed. Immediately after the final key had been pressed, a screen that lasted for 500 msec appeared with the following instruction: “Next Trial”.

The four possible letter pairs were AA, BB, AB and BA. Participants were instructed to initiate their response as quickly as possible once the letter pairs appeared. Trials were presented in a random order, and each condition consisted of 20 trials. In total there were 40 trials for no shift (i.e., same letter in the pair) and 40 trials for shift (i.e., different letters in the pair) conditions.

The RT established for the cognitive task was the time that the participant required to respond to the second stimulus of each trial upon presentation. For the motor task, it was the time required to press the 4^th^ key of the sequence, starting immediately after the pressing of the third one. For both tasks, only correct responses to the entire trial were submitted for further analysis. The remaining responses were considered errors, and their RTs, were not included in the estimations per sessions. Table 1 and 2 shows the number of errors, for the cognitive and the motor task, respectively. The percentage of correct responses for each task is also shown on the same tables.

### tDCS Parameters

Two regions were selected as cortical targets: DLPFC (F3) [24], [45] and M1 (C3) [46].

The stimulation was delivered by a battery driven Eldith Stimulator DC+ and consisted of 15 min of 1 mA (15 sec ramp up and down) applied by 35 cm^2^ saline soaked sponge electrodes (current density of 0.029 mA/cm^2^). The active electrode was placed over F3 or C3 in a 10–20 electrode system [47], while the reference was placed on the contralateral supraorbital area.

The type of tDCS was balanced between subjects, and the wash out was at least 90 minutes between sessions. We used this wash-out period based upon previous data [48].

To balance the stimulation to prevent order effects on the task, there were three sequences (with five participants each per site) that were applied: 1- anodal, sham and cathodal; 2- sham, cathodal and anodal; 3 – cathodal, anodal and sham.

When the wash-out period was only 90 minutes between the first and the second session, the sham condition (second sequence described above) was performed first to prevent carry over effects to one of the posterior active conditions. The third session for that participant was delivered 24 hours later. All the other sequences (1 and 3) were performed with a wash-out period of 24 hours. The sham condition was performed with only 15 sec ramp up and down (the electrodes remained on the head for the entire 15 min), with an anodal electrode configuration.

### Experimental Design

**Figure 5:**
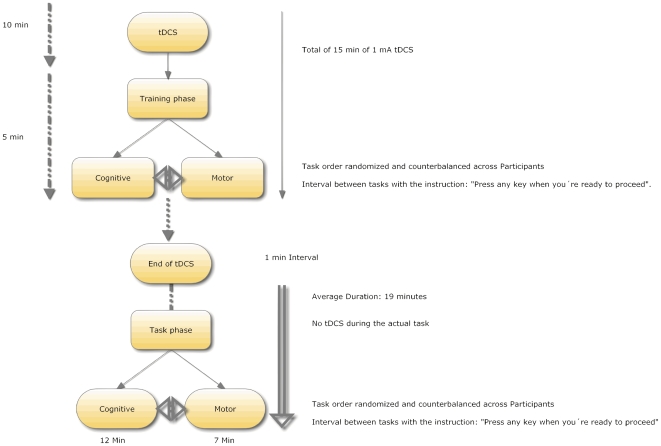
Schematic Representation of the experimental design used in this experiment.

Fifteen participants were randomly assigned only to one site, namely left DLPFC or left M1. The stimulation started prior to the training phase and the last 5 min were delivered while the participants were performing the 5 min of training, because this could improve learning related NMDA receptor strengthening [49], [50] with longer lasting effects than training alone [51]. No tDCS was applied during the actual task, as the aim was to test the after effects of the polarity in task performance. The cognitive and the motor experiment were collected in the same session, and the order in each they were performed was randomized and counterbalanced across participants and sessions, in order to prevent order effects due to possible task difficulty differences. The cognitive task had an average duration of 12 min, while the motor task had an average duration of 7 min. The order in which each task (cognitive or motor) was performed was fully randomized and counterbalanced across participants and sessions. Because the objective of the study was to test the aftereffects of tDCS in set shifting, both tasks were performed “offline” (i.e. with no tDCS during the actual task performance) (as depicted in Fig. 5).

### Data analysis

General linear model analysis was used. To test the effects of tDCS on the speed of processing and irrelevant set filtering in the cognitive task, mixed model ANOVAs were used, with three within subject levels as TDCS (anodal, sham and cathodal), two within subject levels as SHIFTING (No Shift and Shift), three within subject levels as FILTERING (Alone, Neutral, Incongruent) and two between subjects levels as SITE (DLPFC and M1).

For the tDCS effects on the speed of processing in the motor task, mixed model ANOVAs were performed, with three within subject levels as TDCS (anodal, sham and cathodal), two within subject levels as SHIFTING (No Shift and Shift) and two between subjects levels as SITE (DLPFC and M1).

For error analysis in the cognitive task, mixed model ANOVAs were performed, with three within subject levels as TDCS (anodal, sham and cathodal), two within subject levels as SHIFTING (No Shift and Shift), three within subjects levels as FILTERING (Alone, Neutral and Incongruent), and two between subject levels as SITE (DLPFC and M1). For the motor task similar analysis was performed, with three within subject levels as TDCS (anodal, sham and cathodal), two within subject levels as SHIFTING (No Shift and Shift) and two between subject levels as SITE (DLPFC and M1).

For tDCS effects on shift cost (i.e., Reaction Time (RT) difference between shift and no shift trials) on both tasks, mixed model ANOVAs were performed, with three within subject levels as TDCS (anodal, sham and cathodal) and two between subject levels as SITE (DLPFC and M1).

Although this study was underpowered to include all the factors in a full multifactorial analysis, we conducted an exploratory analysis in order to investigate the possible interaction between TASK and TDCS. Therefore two multifactorial analysis of TDCS (with three levels), SHIFTING (No shift and Shift), TASK (with two levels cognitive and motor) and SITE as between subject factor (DLPFC and M1) were performed, one for the RTs and the other for the percentage of correct responses (because of the number of trials between the two tasks was different). A multifactorial analysis of TDCS (with three levels), TASK (with two levels cognitive and motor) and SITE as between subject factor (DLPFC and M1) was performed for shift costs.

When sphericity was not met, the Greenhouse-Geisser correction was applied to degrees of freedom in all cases, with the corrected probabilities and partial eta-squared (η_p_^2^) statistic reported. Post hoc comparisons of the mean values were carried out by paired multiple comparisons (adjusted to Bonferroni) when the ANOVAs revealed significant effects due to the factors and their interactions. The criterion for statistical significance was established at p<.05. All statistical analyses were performed with IBM SPSS for Windows (version 19.0.1).

Data are presented as Mean (M) and SEM (SE) (CI 95%). In order to deal with possible outliers, there was an established cut off point for each task: responses with scores over 2000 msec in the cognitive task were considered outliers, as well as scores over 700 msec for the motor task (this represents less than 2.5% of the total number of scores).
